# Supplementary figures and images for: Effects of rebound exercises on balance and mobility of people with neurological disorders: A systematic review
Source: PLoS One. 2023 Oct 5;18(10):e0292312. doi: 10.1371/journal.pone.0292312 (PMC10553300; doi:10.1371/journal.pone.0292312)

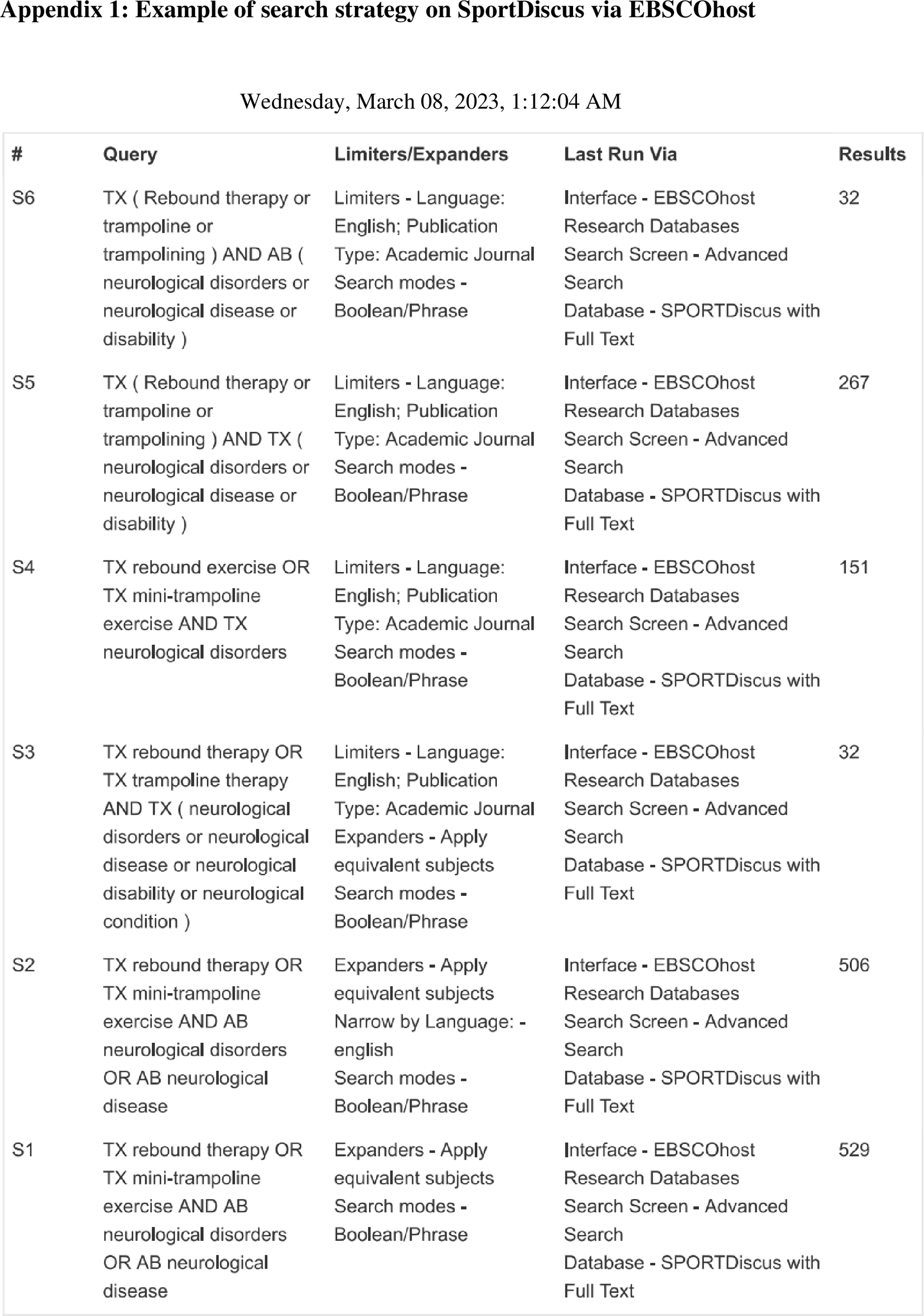

Supplement: S1 Appendix — (TIF) [file pone.0292312.s001.tif]

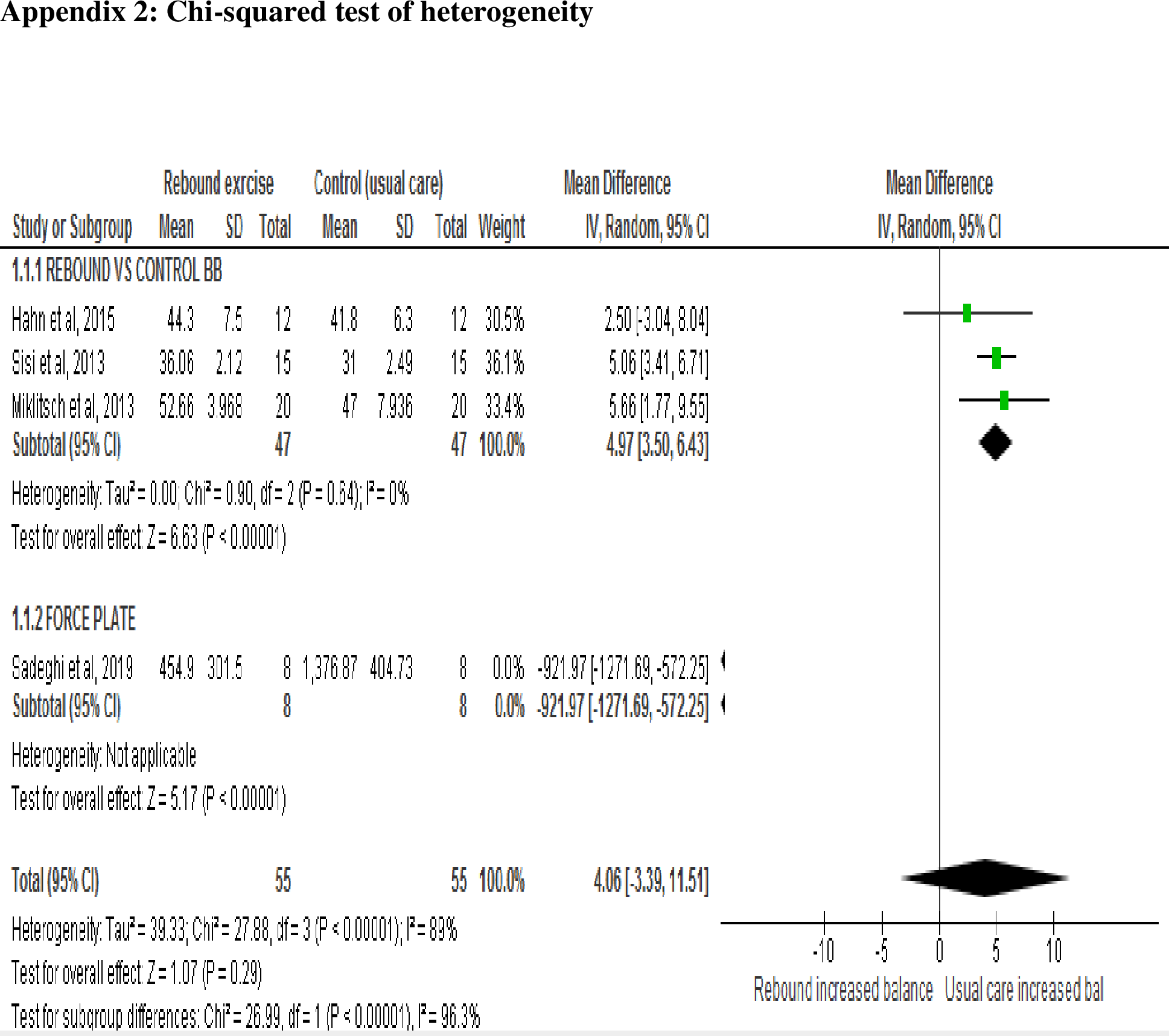

Supplement: S2 Appendix — (TIF) [file pone.0292312.s002.tif]
